# Allocation of home care services by municipalities in Norway: a document analysis

**DOI:** 10.1186/s12913-017-2623-3

**Published:** 2017-09-22

**Authors:** Solrun G. Holm, Terje A. Mathisen, Torill M. Sæterstrand, Berit S. Brinchmann

**Affiliations:** 1 Faculty of Nursing and Health Sciences, Nord University, Storgt 105, 8370 Leknes, Norway; 2 Nord University Business School, 8049 Bodø, Norway; 3 Faculty of Nursing and Health Sciences, Nord University, 8455 Stokmarknes, Norway; 4 Faculty of Nursing and Health Sciences, Nord University, 8049 Bodø, Norway

**Keywords:** Home health nursing, Practical assistance, Support contact, Community, Healthcare delivery, Aging

## Abstract

**Background:**

In Norway, elder care is primarily a municipal responsibility. Municipal health services strive to offer the ‘lowest level of effective care,’ and home healthcare services are defined as the lowest level of care in Norway. Municipalities determine the type(s) of service and the amount of care applicants require. The services granted are outlined in an individual decision letter, which serves as a contract between the municipality and the home healthcare recipient. The purpose of this study was to gain insight into the scope and duration of home healthcare services allocated by municipalities and to determine where home care recipients live in relation to home healthcare service offices.

**Methods:**

A document analysis was performed on data derived from 833 letters to individuals allocated home care services in two municipalities in Northern Norway (Municipality A = 500 recipients, Municipality B = 333 recipients).

**Results:**

In Municipality A, 74% of service hours were allotted to home health nursing, 12% to practical assistance, and 14% to support contact; in Municipality B, the distribution was 73%, 19%, and 8%, respectively. Both municipalities allocated home health services with no service end date (41% and 85% of the total services, respectively). Among recipients of “expired” services, 25% in Municipality A and 7% in Municipality B continued to receive assistance.

**Conclusions:**

Our findings reveal that the municipalities adhered to the goal for home care recipients to remain at home as long as possible before moving into a nursing home. The findings also indicate that the system for allocating home healthcare services may not be fair, as the municipalities lacked procedures for revising individual decisions. Our findings indicate that local authorities should closely examine how they design individual decisions and increase their awareness of how long a service should be provided.

## Background

In Norway, as in many other countries, the goal is for disabled residents who require home healthcare services to remain at home as long as possible before moving into a nursing home. Home healthcare services are defined as the ‘lowest level of effective care’, LEON, in Norway [1]. Home healthcare service became part of the municipalities’ public health service in 1972, and LEON was introduced in a White Paper in 1974. Since then, it has been one of the basic principles of Norwegian health care policy [2].

According to the Health and Care Services Act, Norwegian inhabitants have a legal right to home healthcare services, irrespective of age, gender, socioeconomic status, or other differences [3]. These services are organized, managed, and primarily financed by Norwegian municipalities, and this approach to healthcare is called the Scandinavian or Nordic model [4]. Norway, which has approximately 5 million inhabitants, is divided into 428 municipalities. The smallest has 200 inhabitants, and the largest has 658,390, and approximately 55% of the municipalities have fewer than 5000 inhabitants.

Home healthcare services are responsible for performing various services, but in this study, we specifically focused on home health nursing, practical assistance (formerly referred to as home help), and support contact. According to Fjørtoft [5], home health nursing provided by registered nurses and licensed practical nurses is a comprehensive service that includes rehabilitative, therapeutic, and assistive home healthcare, in addition to nursing. This service is administered to people who require home healthcare services for either a short or long period as a result of illness, impaired health, old age, or other factors. The practical assistance provided by licensed practical nurses and home care aides includes help with personal and instrumental activities of daily living (PADL and IADL, respectively). Individuals who are completely dependent on practical or personal help to manage their daily activities are entitled to assistance. They pay a fee to cover part of the expenses for these services, and the municipality covers the remainder of the cost. Support contact is a service provided to individuals and families who, as result of disability, age, or mental health problems, require personal assistance to avoid isolation or to live a socially active life. Support contacts’ primary task is usually to help these individuals engage in meaningful leisure activities. They do not replace professionals or volunteers; instead, they are meant to provide a supplementary service that is paid for by home healthcare services. The role of support contacts is comparable to that of voluntary workers in other Western countries [6–8].

In Norway, home health care services are organized in two different ways. We use a traditional organizational model that does not distinguish between providing and administering the services and a purchaser-provider model that splits the provision and administration of home healthcare services into two separate units. Medium and small municipalities combine these two models. Nurses in the corresponding sectors of the home healthcare services assess applicants and make draft decisions, which are approved by the purchaser unit. The purchaser unit sends reply letters to the applicants for the granted services.

All individuals with special-assistance needs due to illness or disability can apply for home healthcare services in the municipality in which they are living or staying. Depending on the services applied for, it may be necessary to obtain information from the applicant. Typically, nurses conduct a home visit to collect the necessary information about the applicant. To collect the necessary information, nurses use a standardized assessment form, IPLOS, which all municipalities in Norway are required to use. IPLOS is an acronym for “Individbasert pleie- og omsorgsstatistikk”, or “Statistics linked to individual needs for care” [9, 10]. Using the IPLOS form, the nurse assesses and records the level of help required for daily housework, food and goods supply, personal hygiene, dressing/undressing, toilet routines, eating, walking around the house, walking around outside, taking care of one’s own health, memory, communication, daily life decisions, social activities, controlling one’s own behavior, vision, hearing, and whether the applicant receives help from next of kin. The Norwegian Directorate of Health has written a guide on how to complete the IPLOS form [10]. The IPLOS score reflects what health and care services the applicant needs and receives. The Norwegian Association of Local and Regional Authorities has developed a reference guide with advisory information about the amount of time required to help perform each activity [11]. An example of a time allotment outlined in these recommendations is 30 min for morning care (e.g., toileting), 20 min for wound dressing, 10 min for compression stockings (on/off), and 30 min for showering. These estimates are intended to be used only as a starting point. The municipal administration of home healthcare services then uses this information to make an individual decision about the type and scope of the services the applicant requires. According to Otnes and Haugstveit [12], almost all requests for home healthcare services in Norway are granted (99%). Table 1 provides two examples of individual decisions.

**Table 1:** Examples of how individual decisions are formulated based on individual decisions from the dataset

Individual Decision Example 1 The municipality grants (recipient’s name) 9 h of home health nursing per week starting 01.01.08. The assistance includes drug administration 3 x daily, blood glucose measurements × 3 daily, showering × 1 per week and assistance with putting on elastic stockings daily. You will also receive help with eye drops daily.				
Individual Decision Example 2 The municipality grants (recipient’s name) home health nursing 14 h, 45 min per week. The help will include:				
Measures	Number		Hours	Note
Other measures	3	daily	0:15	Help with meals
Dressing and undressing	2	daily	0:30	Morning care and evening care
Shower	2	weekly	01:00	
Medication	1	weekly	00:30	Fill and deliver the dosette box

Individual decision, referred to as “enkeltvedtak” in Norwegian, has a legal status regarding the rights or duties of individuals, for which special legal rules apply. The individual decision acts as a municipal contract with the applicant that outlines the types of assistance and healthcare deemed necessary for the applicant to remain at home [13–16]. Under the Public Administration Act, applicants for home healthcare are entitled to a written reply with an individual decision issued by the municipality within 30 days [14], and the home care services’ offices receive copies of these letters. The content of the individual decision letters serves as the basis for planning and implementing home healthcare services in collaboration with the individuals who receive them.

To ensure the provision of fair services, the Norwegian Directorate of Health [8] has published a guide for municipalities regarding procedures and documentation in nursing and home healthcare. It describes the requirements for the formulation of individual decisions and states as follows:

When allocating services it has to be clear in the decision letter to the recipient what they can expect of services. This decision letter should as far as possible be designed in such a way that the recipient knows which services are to be provided, their scope, and when they will be given ([8], p. 49).

Furthermore, in an audit report, the Norwegian Board of Health Supervision [17] indicates that the decisions may be regarded as municipalities’ “information label” on the home healthcare services granted.

The Norwegian Directorate of Health [8] clarifies that service provision should be adapted to individual service needs based on individual assessments. If municipalities have different standards for service allocation, conflict can easily arise with regard to the process of individual assessment and individualized services. Nonetheless, an individual’s service provision at any given time must not be less comprehensive than or otherwise fail to meet the acceptable minimum standard level of care.

Prior studies on the delivery of home healthcare services have focused on how employees experience the delivery of home healthcare and whether there is enough time allocated to the required tasks [18–22]. However, we have found no studies that directly analyze documents describing what home healthcare services are allocated by municipalities. This study contributes to a more complete understanding of the allocation of home healthcare services in Norway.

The aims of this retrospective, descriptive study were to gain insight into the information about healthcare services municipalities included in individual decisions. The research questions were as follows:

1. What type of home healthcare services do municipalities allocate, and how do the services differ between the two observed municipalities?
2. What combinations of home healthcare services do home care recipients receive?
3. What is the duration of the services allocated?
4. Are there differences in the scope of services associated with where home healthcare recipients live?

## Methods

This study is part of an innovative user-driven project called the “Development and Maintenance of Good Quality Services to Home Care Recipients in Open Care,” conducted by the Centre for Development of Home Healthcare Services in Nordland in two northern Norwegian municipalities. The aims of this current study were to reveal the scope of the provision of home healthcare services in municipalities and to identify the requirements to address future challenges. The two municipalities that participated in this project agreed to provide information on home healthcare services. These municipalities were selected because they are located in rural regions and are large enough to provide a sufficiently broad set of observations. Moreover, they provide breadth in data because they have different characteristics in terms of where people live and the organization of home healthcare services.

We used document analysis to gain insight into the services home care recipients receive from municipalities. Because documents were the main source in the investigation, we followed the general rule that all relevant documents be examined and analyzed for the time period specified [23–25].

### Participant selection

**Fig. 1:**
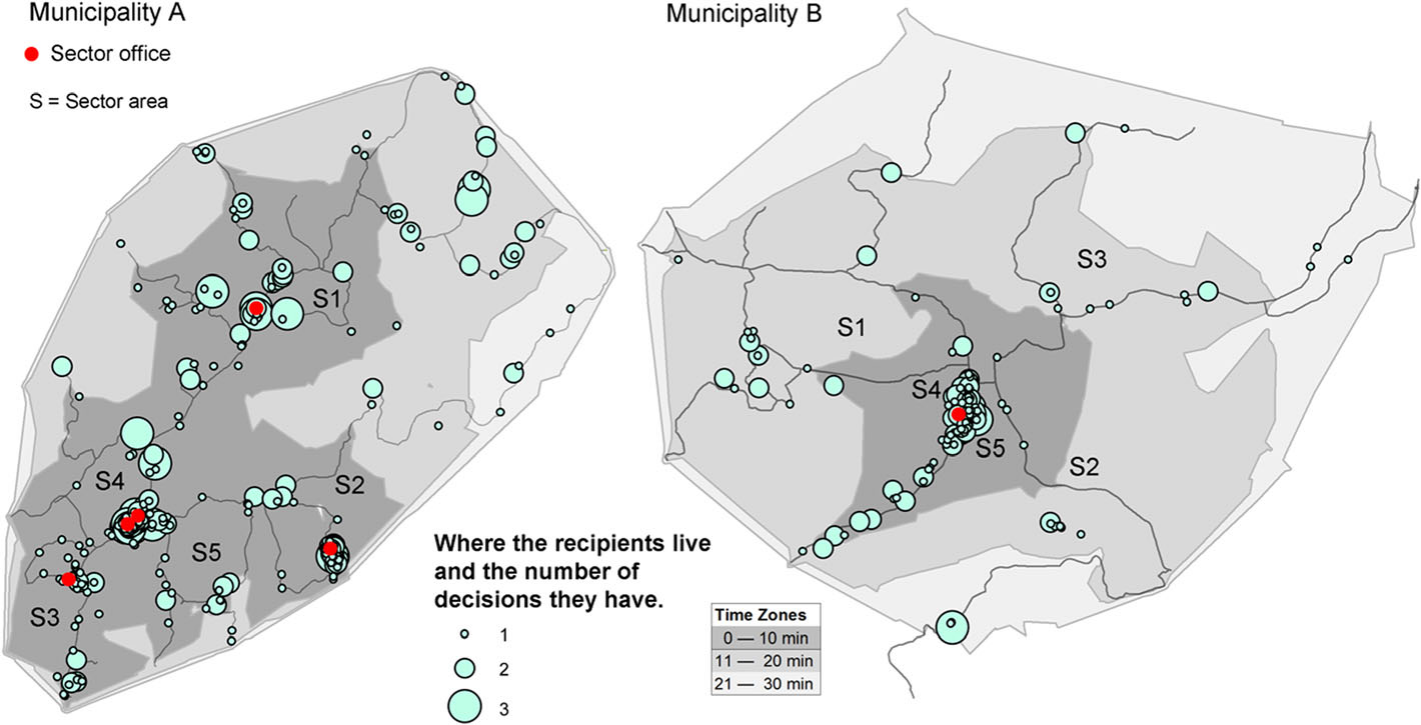
Location of home care recipients, showing the number of individual decisions each recipient has

The sample included home healthcare services in two municipalities (A and B) located in rural northern Norway. Municipality A has a total land area of 405.58 km^2^ (156.60 mile^2^), and Municipality B has a total land area of 698.22 km^2^ (269.58 mile^2^). Although the two municipalities have a similar number of inhabitants, the settlement patterns differ due to geography. In addition to rural and remote areas, Municipality A has four smaller centers, and Municipality B has one (Fig. 1). In 2012, Municipality A had a population of approximately 10,800, and Municipality B had a population of approximately 10,100. That year, 16% of residents in Municipality A and 13% of those in Municipality B were 67 years or older (retirement age in Norway). In both municipalities, home healthcare services are divided into five sectors. Municipality A has three smaller centers, including a sector office and a community center with two sector offices; in Municipality B, all sector offices are located at the community center. The smaller centers in Municipality A have a grocery store and a postal service. In Municipality B, citizens must travel to the municipal center to food shop in a grocery store. In both municipalities, home healthcare recipients live within a 30-min drive of the home care sector offices [18].

### Data sources

The data sources comprised copies of all written individual decision documents outlining the recipients’ home healthcare services to those who received the services this particular week in December 2012. This month was randomly selected, and because the demand for home healthcare services is more or less constant throughout the year, the selection of the study period was not expected to influence the results.

The criterion for document selection was that the document contained individual decisions pertaining to home health nursing, practical assistance, and/or support contact in December 2012. Each sector office set up a list of recipients who received services, and based on these lists, we obtained copies of the decisions stored in the electronic journal system. A total of 833 individual decisions were collected, 500 from Municipality A and 333 from Municipality B.

### Coding and data analysis

Each individual decision letter was registered with a unique combination of a serial number and the registered home healthcare service number. This method was used to identify recipients who received more than one service. To analyze the documents, we developed a coding system based on the content requirements for decisions provided in the Administrative Procedures and Documentation for Nursing and Care Services manual [8] and the Quality in Nursing and Care Services manual [26]. In the codebook, we included a list of variables to examine and a coding scheme. The services and scope of services were divided into three main categories: home health nursing, practical assistance, and support contact. Each of these services was allotted a certain number of hours and minutes per week. We also recorded the dates of service startup and service termination, which were outlined in an additional category, duration of services. Under personal information, we recorded recipients’ gender and year of birth.

For the analysis of where users live, a geographic information system (GIS) was used. A GIS for home service analysis requires geographic data of available addresses in the municipality and data on the municipality’s road network, including the speed limit on each road trip. These data were made available by participating municipalities. All recipients and sector offices were added to the system and located based on their address (geocoding). The local sector offices and recipients’ addresses were stored in separate data sets. All other information, such as the services recipients received (home care, practical assistance and support contact), were added as attributes. From another study in the main project the distances traveled by staff to each sector were analyzed using GIS [18].

Data extraction was performed by two trained assistants with master’s degrees who were familiar with the terminology and specifically trained to extract relevant information from the decision letters. The registered content included information such as dates and allocated hours and minutes. The documents were split into two piles, and each assistant transcribed the information from her pile. The data reliability was checked by ensuring that 10% of the documents were randomly selected and compared with the registered data [27]. All documents that contained data with large deviations were also compared with the registered data, and all data from the controlled documents were registered as correct.

### Statistical analysis

Data that required geographic reference were analyzed using ArcGIS version 10.2.2 Network Analyst geographical information system (GIS) software. Independent means *t*-tests were performed using Strata version 12 to determine whether the time allocated for home health nursing, practical assistance, and support contact in the sectors differed significantly. Significant deviations are presented in Table 2. The dependent variable was the score (allocated time), and the independent variable was the differentiator of the two scores (municipality or sector).

**Table 2:** Average and median numbers of hours of home healthcare services per week

Municipality A					Municipality B				
	Hours	Average^a^	Median	*SD*		Hours	Average^a^	Median	*SD*
Home health nursing					Home health nursing				
Sector 1 (*n* = 53)	136	2.6	2.0	2.3	Sector 1 (*n* = 47)	93	2.0*	1.0	4.1
Sector 2 (*n* = 60)	287	4.8	2.8	5.6	Sector 2 (*n* = 25)	55	2.2	1.2	2.7
Sector 3 (*n* = 33)	396	12.0*	5.1	37.0	Sector 3 (*n* = 39)	142	3.6	1.2	9.1
Sector 4 (*n* = 73)	265	3.6	2.4	3.9	Sector 4 (*n* = 39)	116	3.0	2.0	3.3
Sector 5 (*n* = 38)	428	10.4*	3.3	30.9	Sector 5 (*n* = 13)	505	38.8**	28.0	34.9
Municipal level (*n* = 257)	1512	5.6	2.4	18.3	Municipal level (*n* = 163)	911	5.6	1.5	14.8
Practical assistance					Practical assistance				
Sector 1 (*n* = 43)	47	1.1	.8	.8	Sector 1 (*n* = 59)	42	.8	.7	0.9
Sector 2 (*n* = 41)	53	1.3	.8	1.5	Sector 2 (*n* = 25)	15	.5	.5	.3
Sector 3 (*n* = 23)	32	1.4**	.8	1.9	Sector 3 (*n* = 26)	16	.7	.6	.2
Sector 4 (*n* = 32)	32	1.0	1.0	0.6	Sector 4 (*n* = 32)	23	.7	.7	.3
Sector 5 (*n* = 39)	71	1.7**	1.0	2.6	Sector 5 (*n* = 12)	65	6.0**	6.5	3.3
Municipal level (*n* = 178)	235	1.3	0.7	1.6	Municipal level (*n* = 153)	161	1.1	.7	1.7
Support contact					Support contact				
Sector 1 (*n* = 21)	65	3.1**	3.0	1.0	Sector 1 (*n* = 7)	20	2.9	2.0	1.6
Sector 2 (*n* = 8)	31	3.9	4.0	1.1	Sector 2 (*n* = 0)	–	–	–	–
Sector 3 (*n* = 10)	49	4.9**	4.0	2.6	Sector 3 (*n* = 7)	20	2.9	2.0	.7
Sector 4 (*n* = 19)	85	3.9	4.0	1.9	Sector 4 (*n* = 2)	4	2.0	2.0	–
Sector 5 (*n* = 9)	57	3.8	4.0	1.6	Sector 5 (*n* = 1)	2	2.0	2.0	–
Municipal level (*n* = 67)	287	4.0	4.0	1.7	Municipal level (*n* = 17)	46	2.7	3.0	1.1

*Note:* ^a^Significant deviations from the mean are indicated by * at the *p <* .10 level and ** at the *p* < .05 level

## Results

The document analysis revealed that in both municipalities, the 80–89 age group contained the most home visit recipients. The second-largest age group differed between the municipalities (Municipality A = 70 to 79 years; Municipality B = 90 to 99 years). In both municipalities, the majority of recipients in all age groups were women (approximately 70%).

All individual decisions fulfilled the necessary requirements detailed by the authorities [8]. Recipients received a written document that outlined the individual decision regarding the scope of the services to be provided and what recipients could expect. These documents did not indicate the time of day the service was to be provided but stated that recipients would arrange the specifics of visits with the sector office.

In both municipalities, the decisions were primarily written in one of two ways (Table 1). In one format, the decision stated the hours granted per week and provided an overview of the services granted to the recipient. In the other format, a detailed description of how often the services should be administered each week was provided, and a certain amount of time was allocated to each type of service.

### Home healthcare services provided by the municipalities

In response to research question #1, the findings on the type and scope of home healthcare services revealed the following: Municipality A allocated 74% of service time to home health nursing, 14% to support contact, and 12% to practical assistance. Municipality B allocated 73% service time to home health nursing, 19% to practical assistance, and 8% to support contact.

The findings regarding the average and median number of hours allocated per week to home health nursing revealed that both municipalities had home care recipients with comprehensive needs (Table 2). The allocation differences are due to variations in allocation decisions made by staff in the municipalities. Two sectors in both Municipality A (Sectors 3 and 5) and Municipality B (Sectors 1 and 5) significantly deviated from one another. In Municipality A, the differences were significant at the *p <* 0.10 level; in Municipality B, they were significant at the *p* < 0.10 level for Sector 1 and at the *p* < 0.05 level for Sector 5. In Municipality A, one home care recipient in Sector 3 was granted 210 total hours per week, and one in Sector 5 was granted 213. In Sector 1 in Municipality B, one home care recipient was granted 28 h, 25 min per week. All other recipients received less than 5 h, 20 min per week. In Sector 5, one recipient was granted 94 h, and one was granted 92 h, 15 min per week. At the municipal level, there was no significant deviation.

In terms of practical assistance, Sectors 3 and 5 in Municipality A and Sector 5 in Municipality B deviated significantly from the others at the *p* < 0.05 level. The sectors in Municipality A each had one recipient who received 10 h or more of practical assistance per week, which increased the average. In Sector 5 of Municipality A, one recipient received 30 min per week, which decreased the average.

Additionally, in two sectors in Municipality A (Sectors 1 and 3), support contact significantly differed at the *p* < 0.05 level. These sectors each had a few recipients who obtained more than 7 h per week, which increased the average.

### Combinations of home healthcare services

**Fig. 2:**
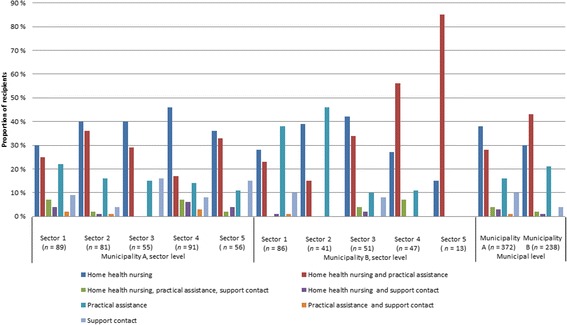
Combination of services allocated in each sector and in each municipality as a whole

The findings concerning the combination of services (research question #2) are shown in Fig. 2. The purpose of Fig. 2 is to show which combinations of services home care recipients have been assigned. We find this figure very informative and have revised the text slightly to highlight the relevance. As Fig. 2 shows, the municipalities differed in that the highest percentage of recipients in Municipality A received only home health nursing, whereas the highest percentage in Municipality B received a combination of home health nursing and practical assistance. For both municipalities, practical assistance was the second most common service, followed by support contact, combined home health nursing, practical assistance, and support; combined home health nursing and support contact; and combined practical assistance and support contact. At the sector level, the greatest percentage of recipients in Municipality A received only home health nursing, followed by combined home health nursing and practical assistance. In Sectors 1, 2, and 4, the third highest percentage of recipients received only practical assistance; in Sectors 3 and 5, the third highest percentage received only support contact. The picture was somewhat different in Municipality B. Specifically, in two sectors (1 and 2), the highest percentage of recipients received only practical assistance, followed by home health nursing. In Sector 3, the highest percentage of recipients received home health nursing, followed by combined home health nursing and practical assistance. In Sector 4, the majority of recipients received combined home health nursing and practical assistance, followed by only home health nursing.

### Duration of services

In response to research question #3, the findings revealed that the service duration ranged from a few days to an indefinite amount of time. In Municipality A, 41% of home care recipients were granted services without an end date, 36% were granted services for 2 years, and 23% were granted services for less than 2 years; among the decisions with an end date, the duration of service ranged from 14 days to 547 days. In Municipality B, 86% of home care recipients received service without an end date. Among decisions with an end date, the service duration ranged from 3 days to 684 days. All individual decisions in both municipalities stated that the overall service would be continuously assessed and would be subject to change if individuals’ assistance needs changed. All individual decisions that included support contact had an end date.

A closer inspection of the service duration revealed that 25% of individual decisions in Municipality A and 7% in Municipality B had “expired,” but the home care recipients were still receiving the service. Only individual decisions about home health nursing had expired. Four individual decisions in Municipality A and five in Municipality B had expired on a date prior to 2006, indicating that these home healthcare recipients had received services for more than 6 years without a formal individual decision. The most outdated individual decision in Municipality A expired in 2005, and that in Municipality B had expired in 2004. Twenty-six individual decisions in Municipality A and 11 in Municipality B had expired between 2006 and 2010. Thirty-six individual decisions in Municipality A and one decision in Municipality B had expired in 2011. In November 2012, 58 and 8 individual decisions had expired in Municipalities A and B, respectively.

### Location of home care recipients

In response to research question #4, the map in Fig. 1 shows that the majority of the home healthcare recipients lived within a 10-min drive of the sector offices (Municipality A = 88%, Municipality B = 84%). Of all the sectors in Municipality A, Sector 1 deviated from this pattern the most. Specifically, only 52% of home healthcare recipients lived within a 10-min drive of the sector office. In Municipality B, 76%, 73%, and 71% of home healthcare recipients in Sectors 1, 2, and 3, lived within a 10-min drive of the sector office, respectively. As shown in Fig. 1, these differences were due to settlement patterns within the sectors. Figure 1 also shows how many decisions each home healthcare recipient received, and it reveals that most recipients of both home health nursing and practical assistance and support contact lived close to the sector office. The exceptions were Sector 1 in Municipality and Sector 2 in Municipality B.

Sector 1 in Municipality A was noteworthy because recipients were more spread out there than in other sectors. Recipients who received 7 h of support contact or more lived near the sector offices, whereas those in rural areas received 4 h or less.

## Discussion

An unexpected finding was the design of many of the decisions (Table 1, Example 2) since the Norwegian Directorate of Health [8] has issued a guide that shows how individual decisions should be formulated. Why are many of the individual decisions not formulated in accordance with the guidelines? One potential explanation is that the most detailed form may be the basis for calculating the hours and minutes per week to be stated in the decision. Since an audit from the Norwegian Board of Health Supervision (17) compares individual decisions with an information label some professional staff think the use of a detailed form is the best way to formulate individual decisions.

The first notable finding is the number of individual decisions without an end date, which may be understood in several ways. One possible explanation is that the examples of individual decisions in the Administrative Procedures and Documentation for Nursing and Care Services document published by the Norwegian Directorate of Health [8] have no end dates, which may lead municipalities to simply follow these guidelines. Because most recipients of home healthcare services are over 70 years of age, this finding could also be based on the expectation that individuals will require services for the rest of their lives. All individual decision letters include a phrase that states that the recipient’s need for home healthcare services will be continuously assessed and that changes will be made if care needs change. Thus, the design of individual decisions requires home healthcare employees to understand the necessity of providing feedback regarding whether there is a need to change or discontinue a recipient’s service. Additionally, individual decisions must be clearer about when a service should be revised or terminated. In the current structure, home healthcare recipients may receive the service for rest of their lives, regardless of whether it is required.

The second key finding is the high number of expired individual decisions that were still in operation. Notably, only decisions about home health nursing had expired. One possible explanation is that home health nursing is the only service that does not involve payment. In contrast, recipients pay a deductible for practical assistance, and a support contact is an external service paid for by home healthcare services. For these services, the deductible payment is controlled, and individuals who work as support contacts receive payment.

It is also possible that missing individual decisions and expired decisions are the result of poor recordkeeping. Although no study has directly examined the contents of individual decisions in association with recordkeeping, a number of studies have examined the quality of home healthcare employees’ electronic patient record (EPR) documentation [28–30]. The results reveal that EPR documentation is frequently incomplete. Consequently, our findings regarding expired decisions appear to be consistent with prior reports about incomplete electronic health records.

Missing individual decisions and expired decisions are proof that employees do not comply with all individual decisions. This is consistent with qualitative research on home healthcare services. When the time allocated in individual decisions is not in line with the time the employees consider necessary to perform proper service, the recipients’ situation takes precedence [21, 31, 32]. Kirchhoff [31] defines services employees provide beyond what is assigned as hidden services. In Denmark, it is called civil disobedience. This research reveals two conflicting rationalities that employees experience with regard to recipients. The leaders of the home service are aware of this problem, and several municipalities in Norway and Denmark are trying to find a new way of managing home healthcare services, which they call “trust reform” [32]. “Trust reform” is based less on control and more on trust and responsibility in employees and their abilities to lead themselves and provide proper services [33].

These findings reveal that employees provide home healthcare services that are not described in individual decisions. This practice is inconsistent with the fundamental ideals of a fair distribution of services and equal access and the notion that recipients with equal needs should be treated equally [34].

### Allocation of home healthcare services and recipient locations

As expected, home health nursing was allocated the most hours in both municipalities. However, Municipality A allocated more hours to support contact than to practical assistance, whereas the opposite was true in Municipality B. The main reason for this difference may be that Municipality B allocated a percentage of hours to PADL as part of practical assistance, whereas in Municipality A, PADL was included as part of home health nursing. Specifically, PADL assistance was described as a service included in Municipality B’s minimum standard for both practical assistance and home health nursing. In Municipality A, PADL assistance was described as a service included in home health nursing. Consequently, municipalities may require home care recipients to pay for PADL; however, this occurs only when the service is not perceived as essential healthcare under the Health and Care Services Act [3]. If PADL is not defined as essential healthcare, then charging for the service is one way that poorer municipalities can improve their financial situation with regard to the provision of home healthcare services. The number of municipalities that charge for PADL in Norway is currently unknown. According to the Norwegian Directorate for Health’s municipality guide, which details procedures and documentation for nursing and care, PADL may be considered either practical assistance or healthcare [8].

In many cases, the allocation of practical assistance is sufficient for people to stay at home longer. In rural areas, many fragile elderly people want to rent municipal sheltered housing (also known as assisted living or housing for care) so that they have easier-to-care-for housing and better access to food stores and other facilities. In Norway, as in certain other Western countries, sheltered housing acts as a supplement and alternative to nursing homes for frail, elderly individuals who require special and extraordinary care [35–37]. In Municipality B, the majority of individuals with comprehensive care needs lived in sheltered housing near the sector office, with the exception of one, who lived approximately 20 min away. In contrast, in Municipality A, many recipients with comprehensive care needs still lived in their original homes. Although the government aims to ensure that as many people as possible remain at home as long as possible, they acknowledge the benefits of individuals with the greatest care needs move into sheltered housing located closer to sector offices. This practice reduces employees’ travel time, allows more time for recipient care, and it makes it easier to follow up with recipients in harsh winter conditions (e.g., blizzards or power failure) [38, 39].

Most individuals who were assigned support contact received only this service. In Municipality A, support contact hours were deliberately assigned to home care recipients with functional impairments living in rural areas. Due to poor transportation, it is necessary to have a support contact with a private vehicle so that he/she can transport recipients to places such as the library, cinema, cafés, and discotheques, to take them on trips, or to accompany them to other events. It is currently unclear why Municipality A assigns more hours to support contact than Municipality B. It may be that Municipality A has more applicants for support contact or that Municipality B has stricter criteria for allocating hours to support contact. According to Westerberg [40], a minority of home care services in Norwegian communities offers support contact. It might also be that municipalities do not make support contact service sufficiently available because it is a free service for recipients or because the municipalities do not consider support contacts to be a valuable service [41].

## Limitations and further research

To our knowledge, this is the first study to perform a document analysis of individual decision letters about home healthcare. Generalizability is limited as data only is collected from two rural municipalities. We consider the findings of our study as a first step getting insight and knowledge about individual decisions, how they are formulated, and follow up by the employees.

Further studies should include more municipalities with research on how municipalities allocate home healthcare services and how individual decisions are followed-up by employees in the sector offices. On the issue of support contact services, these types of services should be studied by researchers to determine what services are needed and given and whether they are effective. This would to give more guidance to municipalities.

## Conclusion

The study has analyzed documents from home healthcare services, focusing on information about healthcare services municipalities allocated in individual decisions. Findings show that even though the individual decisions have expired on date, recipients get home healthcare services. Providing services without an individual decision may not be fair, related to recipients who have an individual decision. The municipalities have insufficient routines for revising individual decisions.

## Practice implications

The implications of our findings for local authorities are that they should closely examine how they design individual decisions and increase their awareness of the length of time a service should be provided. Another practice implication is that the municipalities need better routines for revising individual decisions. Our advice is that the home healthcare services have a formal reassessment of home care recipients and allocation of services twice a year in conjunction with the municipalities revise budget in the spring, and the annual budgetary procedure in the autumn.

## Abbreviations

GIS: Geographical information system
IADL: Instrumental activities of daily living
LEON: Lowest level of effective care
PADL: Personal activities of daily living

## References

[1] Johansen E, Fagerström L (2010). An investigation of the role nurses play in Norwegian home care. Br J Comm Nurs.

[2] Sørbye LW, Norberg A, Hamran T. Frail homebound elderly: basic nursing challenges of home care: a comparative study across 11 sites in Europe. Tromsø: Universitetet i Tromsø; 2009. https://munin.uit.no/handle/10037/1937. Accessed 16 Sept 2017

[3] Ministry of Health and Care Services. Act relating to municipal health and care services, etc. (Health and Care Services Act). http://app.uio.no/ub/ujur/oversatte-lover/data/lov-20110624-030-eng.pdf.

[4] Kemp A, Hvid H (2012). Elderly care in transition - management, meaning and identity at work: a Scandinavian perspective.

[5] Fjørtoft AK (2012). Hjemmesykepleie: ansvar, utfordringer og muligheter. Home Health Nursing: Responsibilities, Challenges and Opportunities.

[6] Johannessen A, Hallberg U, Möller A (2013). Motivating and discouraging factors with being a support contact in the dementia care sector: a grounded theory study. Scand J Disabil Res.

[7] Otnes B (2015). Utviklingen i pleie- og omsorgstjenestene 1994-2013 [developments of community health services 1994-2013]. Tidsskrift for Omsorgsforskning [Journal of Care Research].

[8] The Norwegian Directorate of Health. Veileder for saksbehandling IS 2442. [Guidelines for administrative procedures. IS-2442]. https://helsedirektoratet.no/Lists/Publikasjoner/Attachments/1149/Veileder-for-saksbehandling-IS-2442.pdf. Accessed 29 Aug 2016.

[9] Tøndel G. Administrating disability: the case of “assistance need” registration in Norwegian health and care governance. ALTER - Eur J Disabil Res / Revue Européenne de Recherche sur le Handicap. 2009;3:45–62. https://doi.org/10.1016/j.alter.2008.11.001.

[10] The Norwegian Directorate of Health. Veileder for personell i kommunale helse- og omsorgstjenester, IS-1112 [Guide for personnel in municipal health and care services]. Oslo: The Norwegian Dicetorate of Health; 2006.

[11] The Norwegian Association of Local and Regional Authorities. Brukernåndbok for "FRYD" -verktøy for måling av Forhold mellom Ressurser og Ytelse for Dimensjonering av hjemmetjenesten i kommunen. [User Guide for "FRYD" Tool for Measurement of Relationship between Resources and Service for Dimensioning Home healthcare Services in the Municipalities]. Oslo: KS; 2004.

[12] Otnes B, Haugstveit F. Municipal variation in care services (report no. 44). 2015. https://www.ssb.no/helse/artikler-og-publikasjoner/_attachment/243181?_ts=1506fa1fef8. Accessed 29 Aug 2016. Statistics Norway.

[13] Birkeland A, Flovik AM. Sykepleie i hjemmet. [Home Health Nursing]. Oslo: Cappelen.

[14] Ministry of Justice and Public Security. Forvaltningsloven. [Public Administration Act]. http://app.uio.no/ub/ujur/oversatte-lover/data/lov-19670210-000-eng.pdf. Accessed 22 June 2017.

[15] Molven O (2012). Sykepleie og jus. [Nursing and Law].

[16] Vabø M. Organisering for velferd. Hjemmetjenesten i en styringsideologisk brytningstid [Organization for Welfare]. Norsk Institutt for Forskning om Oppvekst. Velferd og Aldring. http://www.hioa.no/Om-HiOA/Senter-for-velferds-og-arbeidslivsforskning/NOVA/Publikasjonar/Rapporter/2007/Organisering-for-velferd. Accessed 29 Aug 2016

[17] Norwegian Board of Health Supervision. Krevende oppgaver Med svak styring: samlerapport fra tilsyn I 2010 Med kommunenes sosial- og helsetjenester til eldre. Educação). 2011. Complex tasks and services require stronger governance. Oslo: Helsetilsynet. https://www.helsetilsynet.no/upload/Publikasjoner/rapporter2011/helsetilsynetrapport5_2011.pdf. Accessed 29 Aug 2016.

[18] Holm SG, Angelsen RO (2014). A descriptive retrospective study of time consumption in home care services: how do employees use their working time?. BMC Health Serv Res.

[19] Mery G, Wodchis WP, Laporte A (2016). The determinants of the propensity to receive publicly funded home care services for the elderly in Canada: a panel two-stage residual inclusion approach. Health Econ Rev.

[20] Oomkens R, Hoogenboom M, Knijn T (2016). Performance-based contracting in home-care work in the Netherlands: professionalism under pressure?. Health Soc Care Comm.

[21] Sæterstrand T, Holm S, Brinchmann B (2015). Hjemmesykepleiepraksis. [home nursing practice]. Klinisk Sygepleje.

[22] Tufte P, Dahl HM (2016). Navigating the field of temporally framed care in the Danish home care sector. Sociol Health Illn.

[23] Bryman A (2012). Social research methods.

[24] Krippendorff K (2012). Content analysis: an introduction to its methodology.

[25] Syvertsen T (1998). Document analysis in media studies: access, source criticism, issues. The overview of public documents and documents from media institutions (print and web sources).

[26] Norwegian Directorate of Health. [Quality in nursing and care services: guideline manual on quality in nursing and care services for intermediation by Municipal Health and Social Health Services Act]. Oslo: Norwegian directorate of Health; 2004.

[27] Lombard M, Snyder-Duch J, Bracken CC (2010). Practical resources for assessing and reporting intercoder reliability in content analysis.

[28] Gjevjon ER, Hellesø R (2010). The quality of home care nurses' documentation in new electronic patient records. J Clin Nurs.

[29] Sockolow PS, Bowles KH, Lehmann HP, Abbott PA, Weiner JP (2012). Community-based, interdisciplinary geriatric care team satisfaction with an electronic health record: a multimethod study. Comput Inform Nurs.

[30] Turjamaa R, Hartikainen S, Kangasniemi M, Pietilä AM (2015). Is it time for a comprehensive approach in older home care clients' care planning in Finland?. Scand J Caring Sci.

[31] Kirchhoff JW. De skjulte tjenestene - om uønsket atferd i offentlige organisasjoner. Karlstad University: faculty of economic sciences, communication and IT, working. Life Sci. 2010.

[32] Jensen SM, Villadsen K. "Civil disobedience" and conflicting rationalities in elderly care. In: Gubrium JF, Andreassen TA, Solvang P, editors. Reimagining the human service Relationship. EDN. New York: Columbia University Press; 2016.

[33] Blom B, Evertsson L, Perlinski M (2017). Social and caring professions in the European welfare states. Policies, services and professional practices.

[34] Tønnessen S, Førde R, Nortvedt P (2009). Fair nursing care when resources are limited: the role of patients and family members in Norwegian home-based services. Policy Polit Nurs Pract.

[35] Iecovich E (2016). Live-in care workers in sheltered housing for older adults in Israel: the new sheltered housing law. J Aging Soc Policy.

[36] Vlachantoni A, Maslovskaya O, Evandrou M, Falkingham J (2016). The determinants of transitions into sheltered accommodation in later life in England and Wales. J Epidemiol Comm Health.

[37] Ytrehus S (2015). The role of the housing allowance for the elderly in Norway: views of recipients. J Housing Elderly.

[38] Joseph GM, Skinner MW, Yantzi NM (2013). The weather-stains of care: interpreting the meaning of bad weather for front-line health care workers in rural long-term care. Soc Sci Med.

[39] Smallbone C, Staniland K (2011). Care in the community: what would happen if the lights went out?. Br J Comm Nurs.

[40] Westerberg TH (2009). Good services in the dementia-care sector: dementia team, day care, and schools for caregivers.

[41] Johannessen A, Möller A (2012). Why do administrators employ or not employ support contacts? A Norwegian qualitative study. Nordic J Soc Res.

